# Plasmid-encoded gene duplications of extended-spectrum β-lactamases in clinical bacterial isolates

**DOI:** 10.3389/fcimb.2024.1343858

**Published:** 2024-02-26

**Authors:** Annika Sobkowiak, Natalie Scherff, Franziska Schuler, Stefan Bletz, Alexander Mellmann, Vera Schwierzeck, Vincent van Almsick

**Affiliations:** ^1^ Institute of Hygiene, University Hospital Münster, Münster, Germany; ^2^ Department of Cardiology I – Coronary and Peripheral Vascular Disease, Heart Failure, University Hospital Münster, Münster, Germany; ^3^ Institute of Medical Microbiology, University Hospital Münster, Münster, Germany

**Keywords:** antimicrobial resistance, ESBL, plasmid, transposons, gene duplication, antibiotic susceptibility

## Abstract

**Introduction:**

The emergence of extended-spectrum β-lactamase (ESBL)-producing Enterobacteriaceae is an urgent and alarming One Health problem. This study aimed to investigate duplications of plasmid-encoded ESBL genes and their impact on antimicrobial resistance (AMR) phenotypes in clinical and screening isolates.

**Methods:**

Multi-drug-resistant bacteria from hospitalized patients were collected during routine clinical surveillance from January 2022 to June 2023, and their antimicrobial susceptibility patterns were determined. Genotypes were extracted from long-read whole-genome sequencing data. Furthermore, plasmids and other mobile genetic elements associated with ESBL genes were characterized, and the ESBL genes were correlated to ceftazidime minimal inhibitory concentration (MIC).

**Results:**

In total, we identified four cases of plasmid-encoded ESBL gene duplications that match four genetically similar plasmids during the 18-month surveillance period: five *Escherichia coli* and three *Klebsiella pneumoniae* isolates. As the ESBL genes were part of transposable elements, the surrounding sequence regions were duplicated as well. In-depth analysis revealed insertion sequence (IS)-mediated transposition mechanisms. Isolates with duplicated ESBL genes exhibited a higher MIC for ceftazidime in comparison to isolates with a single gene copy (3–256 *vs.* 1.5–32 mg/L, respectively).

**Conclusion:**

ESBL gene duplications led to an increased phenotypic resistance against ceftazidime. Our data suggest that ESBL gene duplications by an IS-mediated transposition are a relevant mechanism for how AMR develops in the clinical setting and is part of the microevolution of plasmids.

## 1 Introduction

Antimicrobial resistance (AMR) is an alarming and urgent One Health problem (Ventola, 2015; Kim and Cha, 2021). AMR allows bacteria to resist and persist even though antibiotics are present. Ever since the first introduction of antimicrobials, the number of resistant bacteria has steadily increased and is becoming a major challenge for healthcare systems worldwide (Davies and Davies, 2010; O’Neill, 2014; Murray et al., 2022). The dissemination of AMR is driven by a combination of different factors such as the extensive use of antimicrobial agents (Browne et al., 2021) as well as selection pressure in almost all environments (Kizny Gordon et al., 2017; Chen et al., 2019). Especially in the hospital setting, AMR is a major concern, as they limit treatment options for serious bacterial infections significantly (Huemer et al., 2020).

Hydrolysis of the β-lactam ring of penicillin, the first discovered antibiotic agent, and their derivates by β-lactamases, is among the most important AMR mechanisms. In 1965, the first plasmid-encoded β-lactamase TEM-1 was discovered, which provided significant resistance in addition to the natural occurrence of chromosomal β-lactamase genes (Datta and Kontomichalou, 1965). Point mutations in the *bla* genes coding for β-lactamases generate the fast-growing family of extended-spectrum beta-lactamases (ESBLs), i.e., TEM-type and SHV-type β-lactamases (Bradford, 2001; Behzadi et al., 2020). ESBLs also provide resistance against newer antibiotic classes such as aminopenicillins and cephalosporins. The most commonly found and clinically relevant ESBL genes are *bla*_TEM_, *bla*_SHV_, and *bla*_CTX-M_ (Liakopoulos et al., 2016).

ESBL-producing Enterobacteriaceae (Bradford, 2001) are of special concern in the healthcare setting, as they are listed as critical in the priority pathogens list by the WHO (Tacconelli et al., 2018). Most ESBL-producing Enterobacteriaceae in the hospital setting can be identified as *Escherichia coli* and *Klebsiella pneumoniae.* For ESBL-producing *E. coli*, an asymptomatic and persistent carriage in the human intestine is well-known and has been increasing in the last decades (Bezabih et al., 2021).

Most AMR genes are coded as accessory genes on mobile genetic elements (MGEs) (Partridge et al., 2018) that can be transferred among bacteria on plasmids via conjugation. This conjugation is possible not only between bacteria of the same species but also beyond species borders within a multispecies bacterial community. Therefore, plasmids are believed to be the key drivers in the development and spread of antimicrobial resistance (Carattoli, 2009). Furthermore, different types of transposons are described as MGEs used to transfer AMR genes (Frost et al., 2005). There, AMR genes are often found as accessory genes, for example, within composite transposons. These transposons are characterized by flanking insertion sequence (IS) coding a transposase, and AMR and virulence genes in between. The transposase uses the IS as recognition sites to transfer the whole transposon from one position in the DNA to another as well as from plasmids to the chromosome if a suitable site is found. Other AMR gene transpositions are carried out by unit transposons, where an IS transposase is only found on one site of the AMR gene (He et al., 2015; Varani et al., 2021).

For many years, the investigation of AMR plasmids has been labor-intensive due to the shortfalls of short-read whole-genome sequencing (WGS) technology. For instance, short-read WGS only covers parts of transposons, which leads to possible misassemblies of these sequence repetitions (Juraschek et al., 2021). Recent long-read WGS offers a significant advantage by enabling the complete coverage of plasmids or the generation of reads that span entire transposons. This capability simplifies the detection of transposons and gene duplications within the sequences.

To increase the understanding of AMR development and spread especially in ESBLs, we analyzed multi-drug-resistant Enterobacteriaceae isolated from clinical samples in more detail by long-read WGS. Although plasmids are key drivers of AMR spread, only little is known about the evolutionary and molecular mechanisms of plasmids harboring AMR genes in the clinical setting. Here, we characterize four cases of ESBL gene duplications on plasmids and demonstrate how these duplications affect the AMR phenotype.

## 2 Material and methods

### 2.1 Clinical setting and bacterial isolates

The University Hospital Münster (UHM) is a 1,300-bed tertiary care center in Münster, Germany, admitting on average 55,000 patients per year. Bacterial samples were collected from hospitalized patients at the UHM for an 18-month time period from January 2022 to June 2023 as part of the routine hospital surveillance of multi-drug-resistant bacteria (MDRBs) according to national recommendations (Bundesgesundheitsblatt, 2012). Screening specimens were cultivated on chromID ESBL Agar (biomérieux, Marcy-l’Etoile, France) and incubated at 36°C ± 1°C for 18 to 24 h. Clinical specimens were cultivated on Columbia Blood agar with 5% Sheep Blood (BD, Heidelberg, Germany; aerobic incubation at 35°C ± 2°C ambient air for up to 3 days), and MacConkey selective agar for Gram-negative bacteria (MacConkey II Agar, BD, Heidelberg, Germany; aerobic incubation at ambient air 35°C ± 2°C for up to 2 days) if applicable. During the study period, >500 MDRB Enterobacteriaceae isolates were identified.

### 2.2 Species identification

Species identification was performed by matrix-assisted laser desorption/ionization time-of-flight mass spectrometry (MALDI-TOF/MS) (MALDI-TOF MS, Biotyper^®^ Sirius one, Bruker, Bremen, Germany) with scores above 2.0.

### 2.3 Antimicrobial susceptibility testing

Antimicrobial susceptibility testing was performed using VITEK^®^2 automated system (biomérieux, Marcy-l’Etoile, France). In addition, Etests^®^ (biomérieux, Marcy-l’Etoile, France) for ceftazidime were performed on Müller-Hinton-Agar (Oxoid, Schwerte, Germany) to determine the minimal inhibitory concentration (MIC). All MICs were interpreted according to the 2023 European Committee on Antimicrobial Susceptibility Testing (EUCAST) clinical breakpoints version 13.1 (EUCAST, 2023).

### 2.4 Whole-genome sequencing

DNA from bacterial isolates was extracted using the NEB Monarch Purification Kit (New England Biolabs, Ipswich, MA, USA) and sequenced on a PacBio^®^ Sequel IIe system (Pacific Biosciences, Menlo Park, CA, USA) as described previously (Effelsberg et al., 2021). A *de novo* assembly approach was used for the raw reads and analyzed using the SMRT^®^ Link software suite v. 10 or v. 11 with default parameters.

### 2.5 Whole-genome data analysis, annotation, and visualization

The phylogenetic tree was generated based on WGS data by the Genome BLAST Distance Phylogeny (GBDP) approach using the Type Strain Genome Server (Meier-Kolthoff and Göker, 2019). For genotyping based on multilocus sequence typing (MLST) and core genome MLST (cgMLST) and to determine the clonal relationship, the respective schemes in Ridom SeqSphere^+^ software version 9.0.9 (Ridom GmbH, Münster, Germany) were used (Jünemann et al., 2013). Isolates were rated as clonal when the distance of their cgMLST allelic profiles was ≤5 alleles. Antimicrobial resistance genes were determined using the NCBI AMRFinderPlus (Feldgarden et al., 2019) implemented in SeqSphere^+^. If duplicated AMR ESBL genes were identified, a more detailed analysis of the respective contigs was performed. Contigs were predicted as plasmids using MOB-Suite v3.1.4 (Robertson and Nash, 2018) and further characterized by pMLST Server 2.0 (Carattoli et al., 2014). Plasmids were completely annotated using DFAST v1.6.0 (including additional scans against TIGRFAM and COG database by NCBI) (Tanizawa et al., 2018). Next, it was searched for genetically matching plasmids with the same ESBL gene but without a gene duplication in the dataset by a mash-based approach (Ondov et al., 2016) implemented in SeqSphere^+^. Isolates containing these plasmids were used as references and were analyzed in the same way.

Progressive Mauve (Darling et al., 2004) was used to compare and visualize the similarity of plasmid sequences and to localize the gene duplication site of the transposable regions. In addition, all mobile genetic elements were annotated using ISFinder (Siguier et al., 2006) and MobileElementFinder with the default parameters (Johansson et al., 2021). Plasmid sequences were manually curated using SnapGene^®^ Viewer (Dotmatics; www.snapgene.com).

## 3 Results

### 3.1 Characteristics of bacterial isolates

We identified four isolates (three *E. coli* and one *K. pneumoniae*) containing more than one plasmid-based ESBL gene copy within the isolates found by our routine molecular surveillance. For comparison, we matched these four isolates with more than one plasmid-based ESBL gene to four additional isolates (two *E. coli* and two *K. pneumoniae*) in the dataset containing genetically the same plasmids with only a single ESBL gene copy (**Supplementary Figures 1–4**). These isolate matches are named pairs I–IV and originate from different or the same patient. Details of the isolates analyzed and the patients’ characteristics are listed in **Table 1**. In total, bacterial isolates of six patients were included in the study: four female patients and two male patients. The median age of patients was 63 (32–94) years. According to their medical history based on the medical reports, all patients suffered from chronic or severe infections (**Table 1**).

**Table 1:** Overview of samples analyzed including origin and patients’ backgrounds.

Plasmid pair	Isolate (patient #)	Specimen	Medical history	Antibiotics prescribed	ST	No. of plasmids detected	ESBL-encoding gene
I	Ec1.1 (1)	Rectal screening	Colorectal cancer with liver metastasis	Piperacillin–tazobactam, vancomycin, micafungin	46	3	*bla*_CTX-M-15_
	Ec1.2* (2)	Urine	Ulcer (stage IV) requiring reconstructive surgery	Ampicillin–sulbactam	69	1	
II	Ec2.1 (3)	Tissue	Recurrent periprosthetic hip joint infection	Ceftazidime, vancomycin, meropenem	95	4	*bla*_SHV-12_
	Kp2.2* (3)				353	4	
III	Ec3.1 (4)	Drainage fluid	Primary sclerosing cholangitis, Klatskin tumor, recurrent episodes of cholangitis	Ceftriaxone	453	3	*bla*_CTX-M-14_
	Ec3.2* (5)	Urine	Cystectomy, recurrent urinary tract infections	Nitrofurantoin and others (unspecified, outpatient prescription)	453	2	
IV	Kp4.1 (6)	Blood culture	Renal transplant, recurrent episodes of pyelonephritis	Cotrimoxazole, amphotericin B	1,634	1	*bla*_SHV-2_
	Kp4.2* (6)	Urine			1,634	2	

Isolates containing ESBL gene duplications are marked with an asterisk.

Ec, Escherichia coli; Kp, Klebsiella pneumoniae; ST, sequence type; ESBL, extended-spectrum β-lactamase.

**Figure 1:**
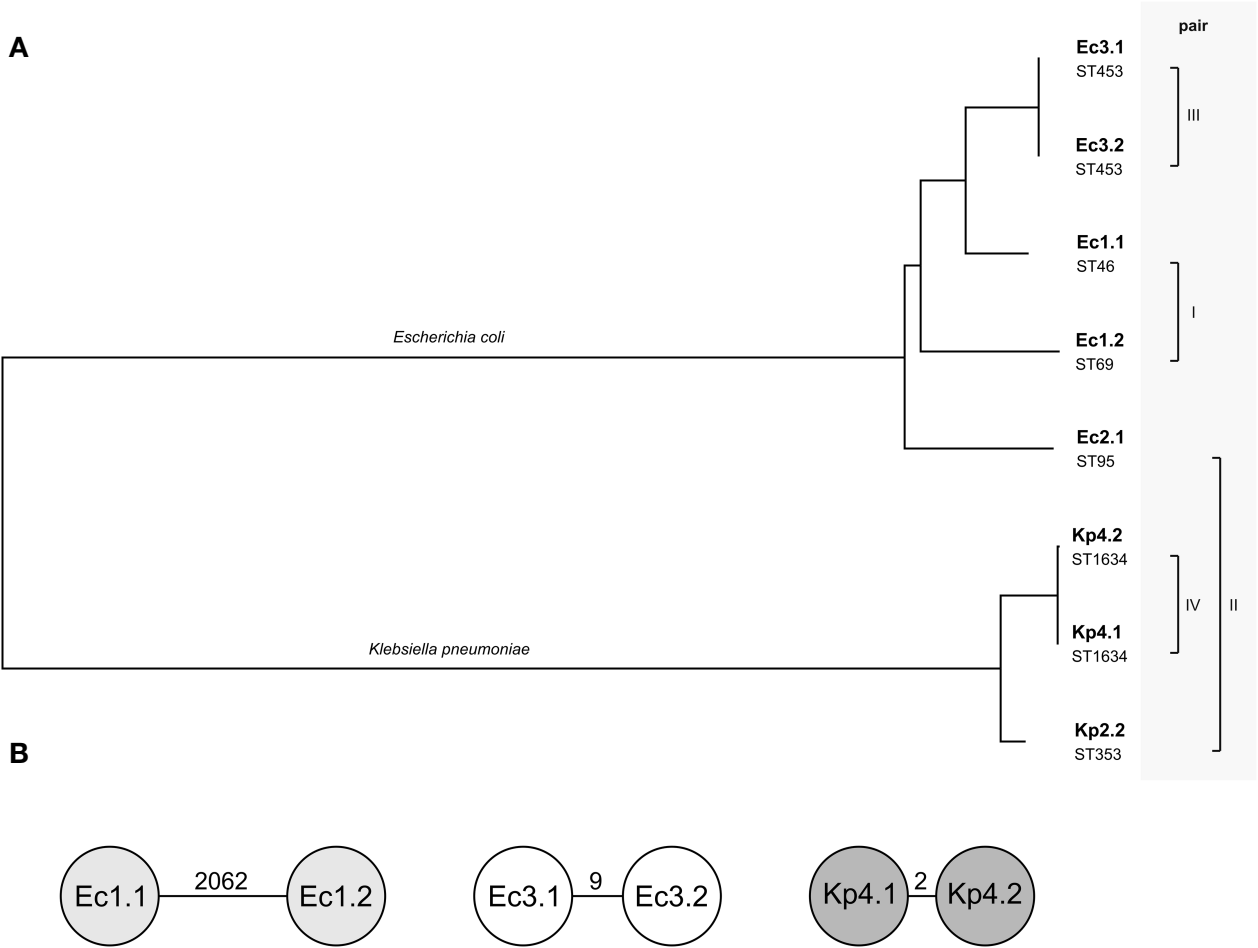
Relationship of the four pairs of isolates included. **(A)** Phylogenetic tree based on whole-genome sequences of the eight isolates calculated by Type (Strain) Genome Server. The branch lengths are scaled in terms of Genome BLAST Distance Phylogeny distance formula. The pairs describe the compared plasmids of the isolates. Below the isolate name, the sequence type (ST) of each isolate is given. **(B)** Detailed relation within the pairs of isolates of the same species using cgMLST. Each circle is a separate genotype based on the allelic profile of up to 2,325 genes for *Escherichia coli* and 2,358 genes for *Klebsiella pneumoniae*, with the parameter “ignoring missing genes in the pairwise comparison”. The number on the connecting lines displays the allelic distance.

The phylogenetic relation of all isolates was calculated based on the WGS data to illustrate the phylogenetic similarity between the isolates in pairs III and IV and the species difference in pair II (**Figure 1A**). In case the matched isolates were the same species, cgMLST typing was performed to determine whether the pairs only share a similar plasmid or in addition have a clonal relation (on the chromosomal level). The isolates of pairs I and III did not show clonality in cgMLST analysis among the *E. coli* isolates (**Figure 1B**). Pair IV was identified as a clonal lineage of *K. pneumoniae* isolates found in one patient (patient 6). Here, the isolates were collected 3 weeks apart in different sample materials (**Table 1**). The similar plasmids of pair II were shared between an *E. coli* isolate and a *K. pneumoniae* isolate that originated from the same specimen of patient 3 (**Table 1**). There was no clonal relationship due to the two different bacterial species.

### 3.2 In-depth analysis of plasmids harboring ESBL-encoding genes

Using the MOB-suite software and AMRFinderPlus tool, we analyzed the plasmids harboring ESBL genes with respect to their sizes, replication and relaxase types, anticipated/predicted mobility, and encoded AMR genes (**Table 2**). Overall, the replication type, the relaxase type, and the predicted mobility of the plasmids were identical within a pair. However, due to the ESBL gene duplication, the plasmid size varied. In addition to the duplicated ESBL-encoding genes, we only found an analog AMR duplication of *qnrS1* in p1_Ec1.2 mediating quinolone resistance. Of note, the similar plasmids p2_Ec2.1 and p2_Kp2.2 are most likely directly related and caused by an intra-host transmission via horizontal gene transfer (HGT) from one species to another.

**Table 2:** Characteristics of analyzed plasmid harboring the ESBL genes.

Plasmid	Size [bp]	Mash distance (hashes)	pMLST	Replication type/s	Relaxase type	Predicted mobility	AMR genes
p3_Ec1.1	72,476	0.0001 (995/1,000)	*[F2:A-:B-]*	IncFIA, IncFIC	MOB_F_	Conjugative	***bla*_CTX-M-15_**, *qnrS1*
p1_Ec1.2	82,307	0.0001 (995/1,000)	*[F2:A-:B-]*	IncFIA, IncFIC	MOB_F_	Conjugative	***bla*_CTX-M-15_**, ***bla*_CTX-M-15_**, *qnrS1*, *qnrS1*
p2_Ec2.1	57,462	0.0015 (940/1,000)	unknown	IncX1, IncX3	MOB_P_	Conjugative	***bla*_SHV-12_**, *bla*_SHV_-family, *qnrS1*
p2_Kp2.2	66,525	0.0015 (940/1,000)	unknown	IncX1, IncX3	MOB_P_	Conjugative	***bla*_SHV-12_**, ***bla*_SHV-12_**, *bla*_SHV_-family, *qnrB19*, *qnrS1*,
p2_Ec3.1	96,364	0.0004 (985/1,000)	unknown	IncI-gamma/K1	MOB_P_	Conjugative	***bla*_CTX-M-14_**
p2_Ec3.2	100,710	0.0004 (985/1,000)	Unknown	IncI-gamma/K1	MOB_P_	Conjugative	***bla*_CTX-M-14,_ *bla*_CTX-M-14_**
p1_Kp4.1	53,023	0.0001 (997/1,000)	*[F-:A13:B-]*	IncFIA, IncR	–	Mobilizable	*arr-3*, *aac(3)-IId*, *aac(6′)-Ib-cr5*, *aadA16*, *tet(D)*, ***bla*_SHV-2_**, *dfrA27*, *sul1*, *sul1*, *qnrB6*
p1_Kp4.2	57,775	0.0001 (997/1,000)	*[F-:A13:B-]*	IncFIA, IncR	–	Mobilizable	*arr-3*, *aac(3)-IId*, *aac(6′)-Ib-cr5*, *aadA16*, *tet(D)*, ***bla*_SHV-2_**, ***bla*_SHV-2_**, *dfrA27*, *sul1*, *sul1*, *qnrB6*

ESBL genes are indicated by bold letters.

ESBL, extended-spectrum β-lactamase.

**Figure 2:**
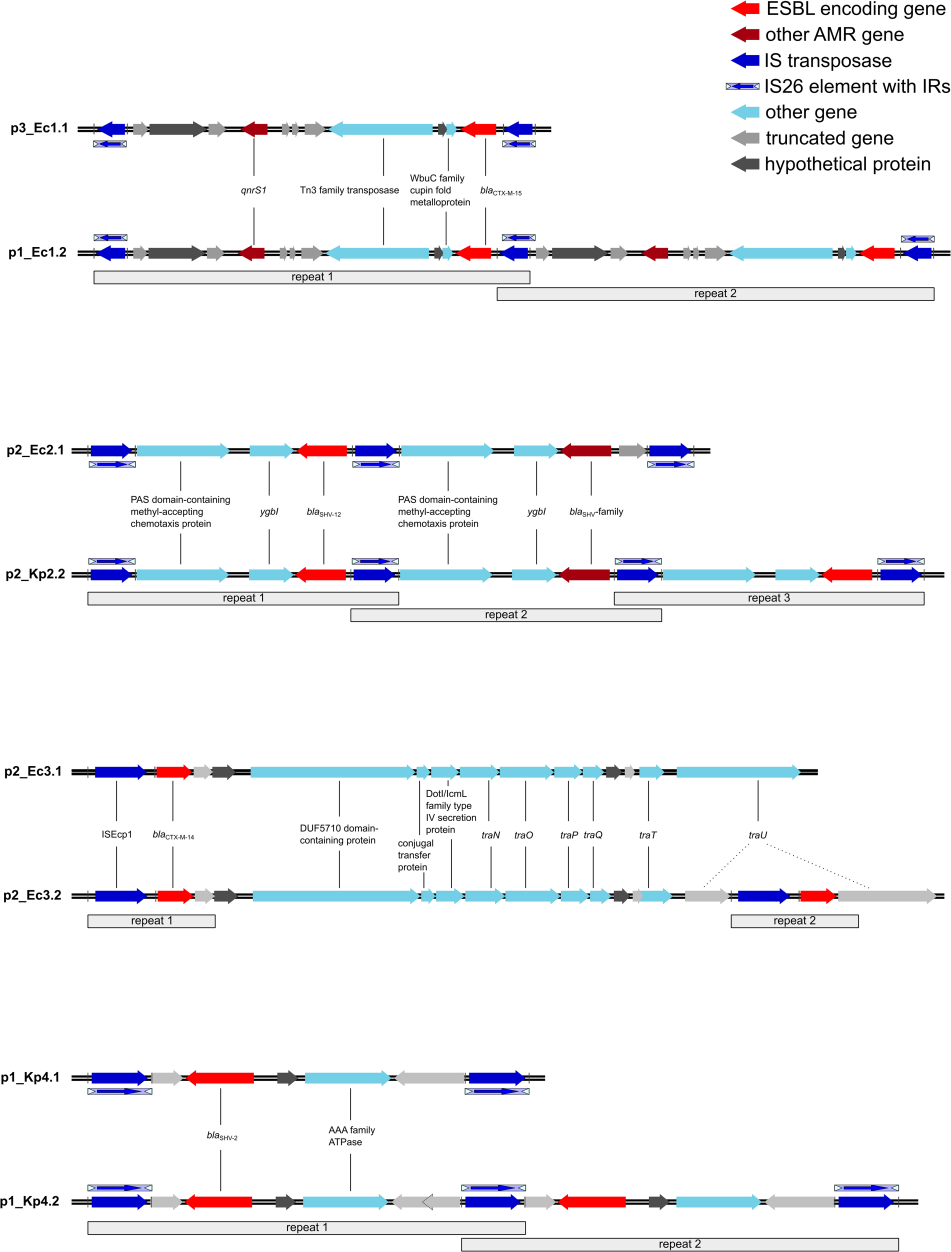
Transposons harboring the ESBL genes and the duplicated regions. All repeated regions harbor the same genes unless indicated otherwise. The *bla*_SHV_-family gene is a mutated variant of *bla*_SHV-12_. Ec, *Escherichia coli*; Kp, *Klebsiella pneumoniae*; ESBL, extended-spectrum β-lactamase; AMR, antimicrobial resistance; IS, insertion sequence; IR, inverted repeats; ESBL, extended-spectrum β-lactamase.

For an in-depth analysis of the plasmids, we annotated the whole plasmids with DFAST and used ISFinder and MobileElementFinder (**Supplementary Figures 1–4**). All duplicated ESBL genes were part of a longer transposable DNA sequence, which was duplicated in full length (**Figure 2**; **Supplementary Table 1**).

In pair I, the duplication occurred in p1_Ec1.2 as a tandem duplication with flanking IS*26* elements (**Figure 2**), notably with three IS*26* elements in comparison to p3_Ec1.1 instead of two flanking IS*26* elements for each duplication. The transposon was classified as a composite transposon and harbors the ESBL gene *bla*_CTX-M-15_ as well as the quinolone resistance gene *qnrS1*. In addition to AMR genes, a WbuC family cupin fold metalloprotein and Tn3 transposase were encoded.

In p2_Ec2.1, the reference plasmid of pair II, we already detected an IS*26* tandem duplication of a transposon harboring the ESBL gene *bla*_SHV-12_. However, there was a difference in the sequence length of one of the transposons caused by an incomplete IS*26* element in the second repeat (**Figure 2**) and a deletion in the ESBL gene (*bla*_SHV-12_/*bla*_SHV_-family). A BLAST analysis revealed a deletion of three nucleotides in the ESBL gene coding originally for leucine 53 in *bla*_SHV-12_. In addition, the transposon contains genes for a PAS domain-containing methyl-accepting chemotaxis protein and for the DNA-binding transcriptional repressor YgbI. While the reference plasmid already shows a duplication, we found a tandem triplication of the transposon in p2_Kp2.2. The coded genes remain the same as in the reference p2_Ec2.1. Nevertheless, one of the three *bla*_SHV_ genes shows a nucleotide deletion as well. The deletion corresponding to threonine 18 in *bla*_SHV-12_ results in a frame shift and an early stop codon, thereby shortening the potentially encoded β-lactamase.

In pair III, we observed another duplication pattern. The ESBL target gene *bla*_CTX-M-14_ in p2_Ec3.2 and the reference plasmid p2_Ec3.1 was carried in an IS*Ecp1*-mediated unit transposon. IS*Ecp1* could be aligned with 100% and 99.94% sequence identities. In addition to the β-lactamase gene, only potential gene residues were transferred within the transposon. The comparison revealed that the point of insertion of the second repeat of the unit transposon in p2_Ec3.2 is the *traU* gene (**Figure 2**).

The plasmid p1_Kp4.2 belongs to pair IV. A tandem duplication of IS*26*-mediated composite transposons could be detected. The one nucleotide difference in the first repeat in p1_Kp4.2 was caused by an insertion in the already truncated oligosaccharide MFS transporter gene as indicated in **Figure 2**. The transposon contains the ESBL target gene *bla*_SHV-2_ and a gene for an AAA family ATPase.

Taken together, IS*26*-mediated duplications were found to be tandem duplications, where one IS*26* element is shared between two transposons. This mechanism was identified in three out of four described cases. An alternative mechanism was observed in pair II. Here, a unit transposon was duplicated in an IS*Ecp1*-mediated mode.

### 3.3 Correlation of phenotypic cephalosporin resistance and ESBL gene copies

To investigate the correlation between the phenotypic antimicrobial susceptibility and the presence of AMR resistance genes, we manually curated the VITEK^®^2 data and used the NCBI AMRFinderPlus to identify matching resistance genes. Furthermore, we used Etests for ceftazidime as a reference substance to determine the cephalosporin resistance more specifically and compare it with the number of ESBL gene copies in the AMR plasmids. In total, AMRFinderPlus annotated 37 different AMR genes within the eight WGS datasets with four to 13 different genes per isolate; including duplications, six to 25 AMR-encoding genes were detected. The lowest numbers of resistance genes were found in Ec1.2 with the duplication of the ESBL gene *bla*_CTX-M-15_ and the *qnrS1*, and *cya*_S352T and *glpT*_E448K. The broadest resistance spectrum showed the three *K. pneumoniae* isolates, with the highest number of AMR genes in Kp4.2. A complete overview of the resistome of the eight analyzed bacterial isolates, categorized into chromosome and plasmid-encoded genes, can be found in the Supplement (**Supplementary Table 2**).

**Figure 3:**
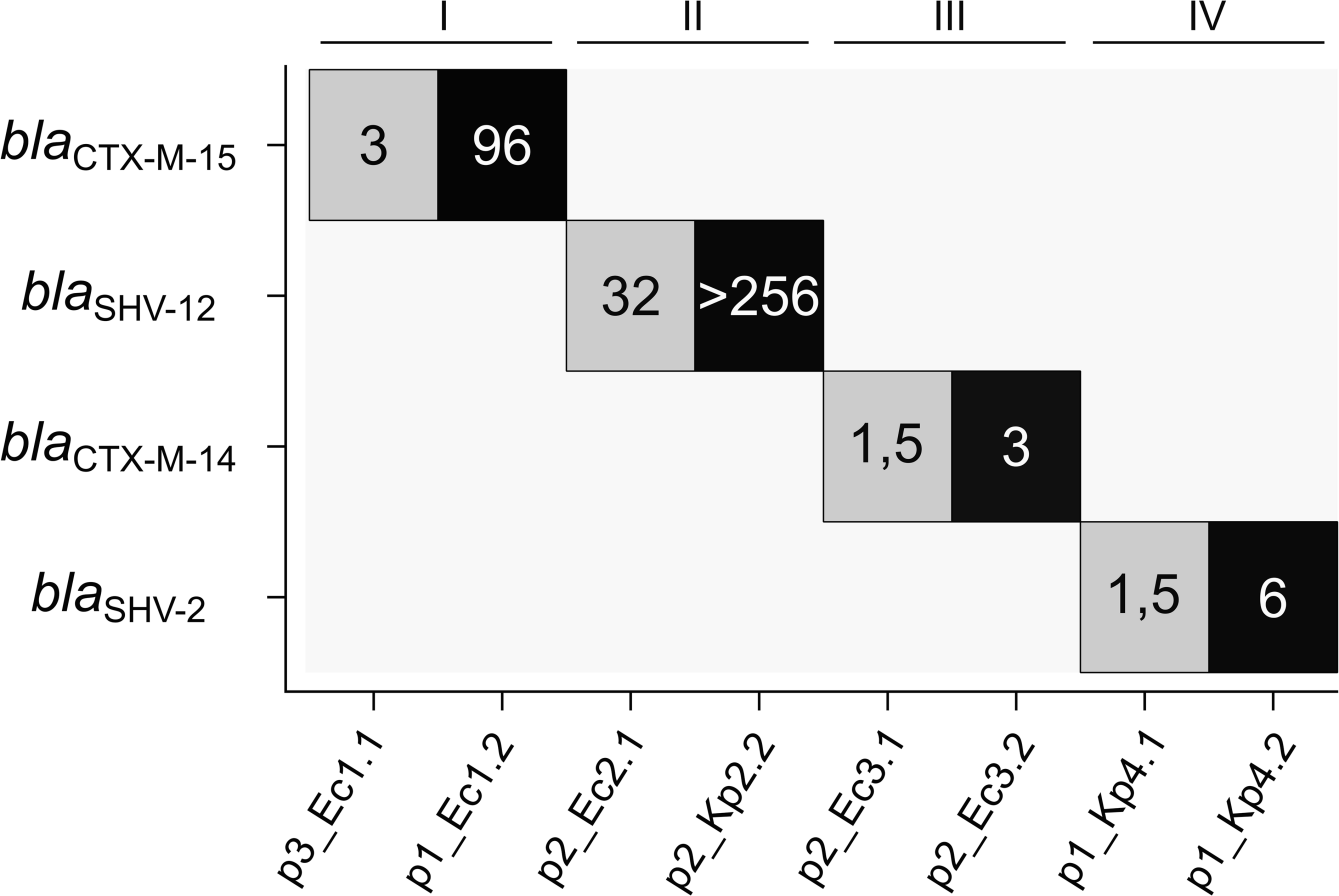
Number of ESBL gene copies in the analyzed plasmids in relation to ceftazidime MIC. ESBL gene copy numbers are color-coded (gray, single copy; black, two copies), and the numbers display the MICs for ceftazidime [mg/L]. ESBL, extended-spectrum β-lactamase; MIC, minimal inhibitory concentration.

The phenotypic resistance pattern of the bacterial isolates was analyzed by comparing up to 12 different antibiotic substances (**Supplementary Table 3**). All isolates were susceptible to carbapenems, and no carbapenemases were detected in the genomic data. In contrast, all isolates were resistant to ampicillin in line with the expression of at least one β-lactamase also found in the genotypic annotation (**Supplementary Table 2**). To determine the MIC of ceftazidime, we performed Etests^®^. The MICs within each pair were found to be more than two times higher for the isolates with the ESBL gene duplications compared to the references (**Figure 3**). The elevated resistance to ceftazidime correlates in all pairs with the higher number of the ESBL target genes.

## 4 Discussion

The emergence of ESBL-producing Enterobacteriaceae is reported worldwide and poses a substantial threat to public health (Murray et al., 2022). There is consensus that plasmids are key drivers for the dissemination of AMR genes such as ESBL genes (Carattoli, 2013). However, little is known about the underlying mechanisms that increase MICs of isolates harboring AMR-encoding genes in the clinical context. Therefore, the aim of our study was to investigate long-read WGS data of clinical bacterial samples with a focus on AMR plasmids and gain insights into the spread of AMR genes in the hospital setting.

We found that ESBL gene duplications occur by transposition events within plasmids and increase the phenotypic resistance against cephalosporins due to the higher gene copy number. Four different cases of gene duplications in plasmids harboring ESBL genes were identified after analyzing the surveillance dataset. Interestingly, all described cases share the IS-mediated transposition with ESBL gene duplication as an *in vivo* evolution event of AMR plasmids and indicate a genotype–phenotype correlation between ESBL gene copy number and increased ceftazidime MIC. Our study highlights the potential of long-read WGS data to better understand AMR and the underlying genetic adaptation mechanisms.

### 4.1 Role of plasmids in AMR spread

All presented cases show gene duplications within the plasmid. The association between plasmids, AMR genes, and a high number of plasmid-borne AMR genes is well known (Vrancianu et al., 2020). Despite the metabolic burden required to maintain a plasmid, most studies could demonstrate that the benefit of the encoded resistance genes is much higher for bacteria or is compensated by additional mutations (Loftie-Eaton et al., 2017; San Millan and MacLean, 2017). This seems plausible for the described cases, as all samples were collected from patients who frequently receive antibiotic treatment. Hence, all isolates have experienced selection pressure.

Interestingly, our study observed highly similar plasmids in different bacterial isolates that showed no genetic relation based on cgMLST (pairs I and III). This could be an example illustrating how plasmids are shared within a community of bacteria with different genetic backgrounds. This HGT is an important mechanism to exchange genetic information known for decades (Thomas and Nielsen, 2005) and well-studied in the adaptive evolution of bacteria (Arnold et al., 2022; Dimitriu, 2022). For the *E. coli* Ec2.1 and the *K. pneumoniae* Kp2.2 (pair II), we assume the HGT of the plasmid harboring the ESBL gene because the species borders have to be crossed. However, it is not possible to determine clearly the chronological sequence of the HGT and the duplication event. On note, even if we discuss predominantly the duplication event, the relation between the compared isolates could also be a loss of a transposable region except in pair IV, where the timeline is clear. Kp4.2 was isolated 3 weeks after Kp4.1 from the same patient.

### 4.2 IS-mediated transposition and other mobile genetic elements associated with ESBL genes

IS-mediated transposition is often observed with accessory genes including AMR genes. All duplication events investigated used this mode of action but with either an IS*26* or an IS*Ecp1* transposition. IS*26* transposition events that could arrange transposons containing AMR genes with only three remaining IS*26* elements were described before including the mechanistic details (He et al., 2015; Harmer and Hall, 2019; Harmer and Hall, 2021). Our data indicate that tandem duplication of the same transposon is also a quite common transposition event as observed in three out of four cases, i.e., in pairs I, III, and IV. In accordance with this observation, a few cases of IS*26*-mediated ESBL gene duplications were described before (Garza-Ramos et al., 2009; Tamamura-Andoh et al., 2021; van Almsick et al., 2022), and also the association of IS*26* and *bla*_CTX-M_ genes was already described in Enterobacteriaceae in a systematic study (Shropshire et al., 2022). Interestingly, neither documented IS*26* transposon with ESBL genes nor a high sequence similarity could be found in the databases of TnCentral (Ross et al., 2021) or Transposon Registry (Tansirichaiya et al., 2019) yet. In the fourth analyzed case (pair II), the transposition was IS*Ecp1* mediated. The detected unit transposon contains a *bla*_CTX-M-14_ gene, which also was found in the *E. coli* isolates of Shropshire et al (Shropshire et al., 2022).

### 4.3 Gene duplications in evolution and correlation to the phenotypic susceptibility

Gene duplication is a common mechanism in evolutionary biology to develop new traits and adaptations (Innan and Kondrashov, 2010; Vosseberg et al., 2021). A new variant to improve resistance by mutation might be observed in pair II annotated as *bla*_SHV_ genes in p2_Ec2.1 and p2_Kp2.2 (**Figure 2**). Both investigated plasmids in this pair have different deletions of nucleotides within one gene copy of the ancestor *bla*_SHV-12_ gene. However, it is unclear whether these mutated genes code for a functional and optimized β-lactamase. Schuster et al. found similar results; they identified a novel *bla*_CTX-M_ gene in this context (Schuster et al., 2022). In that study, the encoded β-lactamase only differed by one amino acid from the ancestor gene that was found in a similar gene array as the new gene. In accordance with this, the evolution of *bla*_SHV_ genes from *bla*_TEM_ with the high sequence similarities underlines this as a plausible mechanism for the generation of novel and better-adapted β-lactamases (Liakopoulos et al., 2016).

In addition, we hypothesize that the increased resistance level to ceftazidime correlates with the number of ESBL gene copies encoded on the plasmid. Only very few reported cases of ESBL gene duplications found this correlation between genotypic and phenotypic resistance so far (Tamamura-Andoh et al., 2021; van Almsick et al., 2022). However, in accordance, this observation has been described before for other AMR genes (Coppi et al., 2020; Han et al., 2021). Nevertheless, increased antibiotic resistance has also been linked to point mutations in resistance genes (Finci et al., 2022). To gain more insights into the genetic resistance patterns of Enterobacteriaceae, more genetic analyses based on studies with higher sample sizes are required.

### 4.4 Study limitations

One limitation of our study is the relatively small number of isolates included in the study. During the 18-month period, four cases of ESBL gene duplications were identified. While this result indicates that ESBL gene duplication is a possible mechanism for developing AMR in the clinical context, higher sample sizes are needed to estimate how frequently this phenomenon happens. Moreover, the comparison of different species and different clonal lineages with variable resistomes has an influence on MIC determination (Girlich et al., 2021). To consider these aspects, we listed additional β-lactamases encoded on other plasmids or integrated into the chromosome for all isolates (**Supplementary Table 1**). Furthermore, the plasmid copy numbers and other regulatory mechanisms were not covered and are out of the scope of this study (Nelson et al., 2017).

## 5 Conclusion

We identified IS-mediated AMR gene duplications as a mechanism to overcome antibiotic selection pressure in the hospital setting and to generate new genetic variants of the ancestor resistance gene. Our results also highlight the genetic potential of Enterobacteriaceae for adaptation and evolution of AMR.

## Data availability statement

The datasets presented in this study can be found in online repositories. The names of the repository/repositories and accession number(s) can be found below: https://www.ncbi.nlm.nih.gov/, PRJNA1030289.

## Ethics statement

Ethical approval was not required for the study involving humans in accordance with the local legislation and institutional requirements. Written informed consent to participate in this study was not required from the participants or the participants’ legal guardians/next of kin in accordance with the national legislation and the institutional requirements.

## Author contributions

AS: Investigation, Data curation, Formal analysis, Software, Visualization, Writing – original draft. NS: Software, Methodology, Validation, Writing – review & editing. FS: Methodology, Validation, Writing – review & editing, Formal analysis, Resources. SB: Validation, Writing – review & editing, Conceptualization. AM: Conceptualization, Data curation, Project administration, Resources, Software, Supervision, Validation, Writing – review & editing. VS: Conceptualization, Data curation, Formal analysis, Methodology, Project administration, Supervision, Validation, Writing – review & editing. VA: Conceptualization, Funding acquisition, Investigation, Project administration, Resources, Supervision, Writing – review & editing.
