# Supplementary material for: Plasmid-encoded gene duplications of extended-spectrum β-lactamases in clinical bacterial isolates
Source: Front Cell Infect Microbiol. 2024 Feb 26;14:1343858. doi: 10.3389/fcimb.2024.1343858 (PMC10925753; doi:10.3389/fcimb.2024.1343858)
Supplement: Supplementary file 1 [file DataSheet_1.pdf]

Supplementary Material

Supplementary Material

1 Supplementary Figures

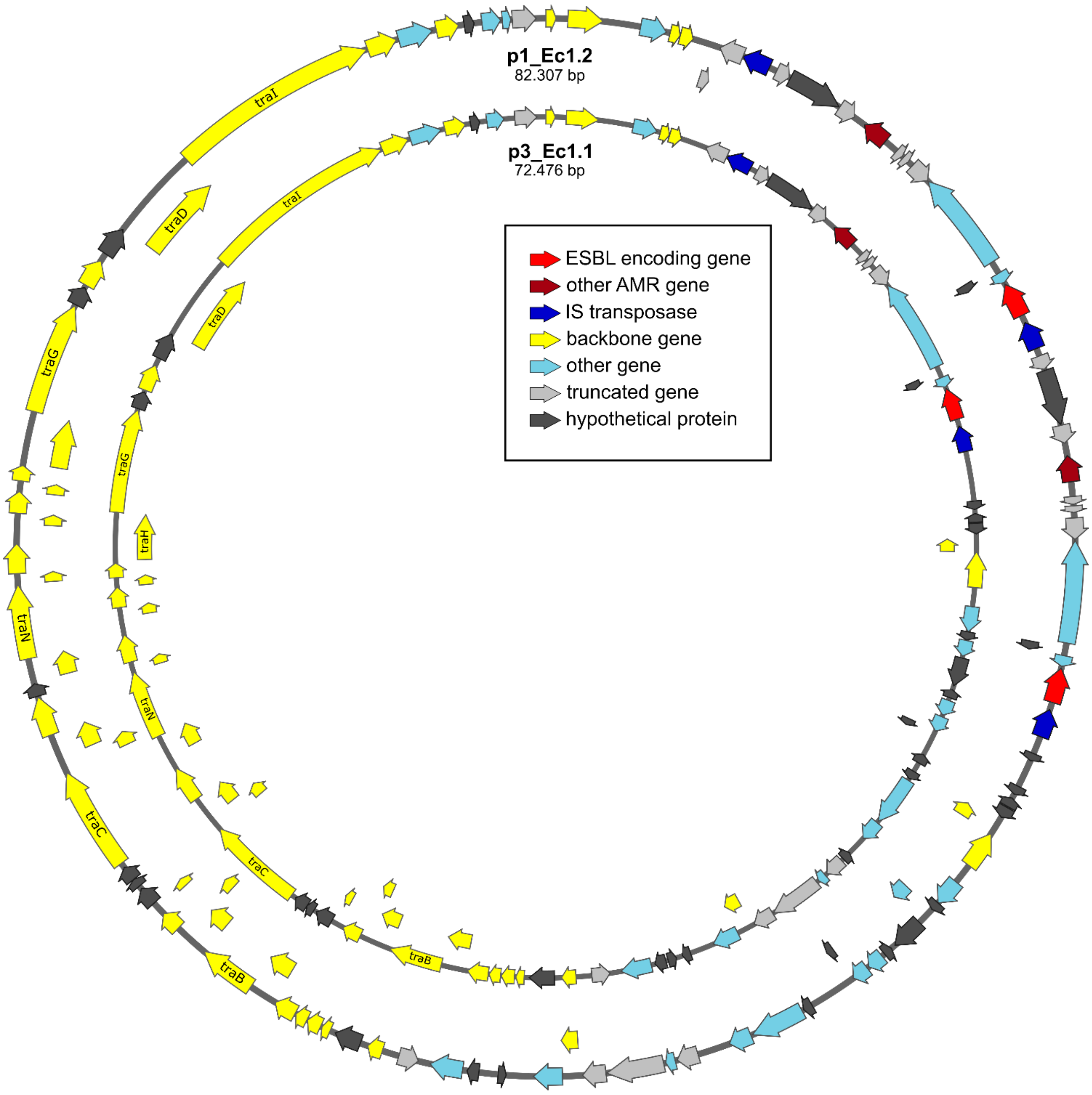

Supplementary Figure S1: Plasmid map pair I, annotated plasmids p3_Ec1.1 and p1_Ec1.2

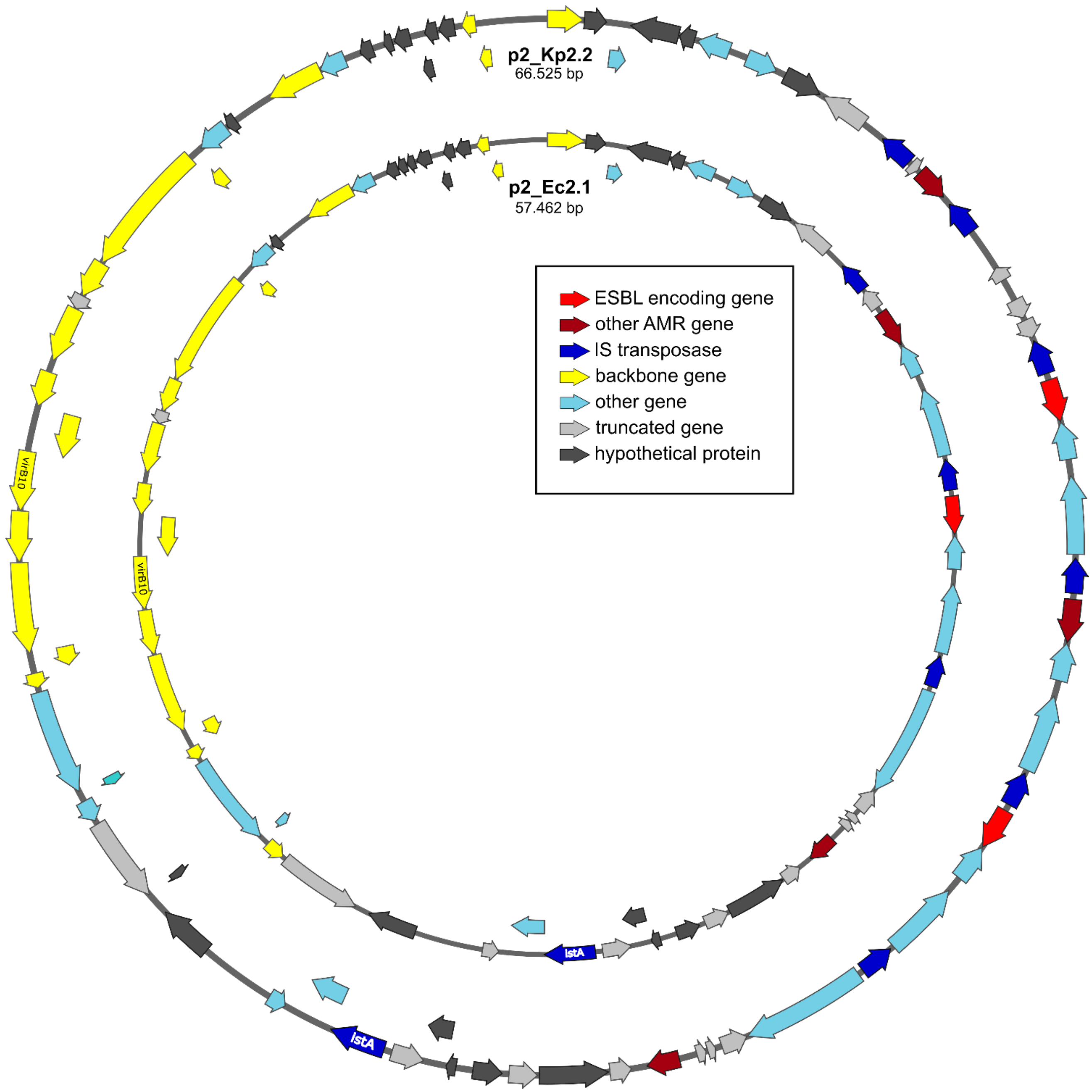

Supplementary Figure S2: Plasmid map pair II, annotated plasmids p2_Ec2.1 and p2_Kp2.2

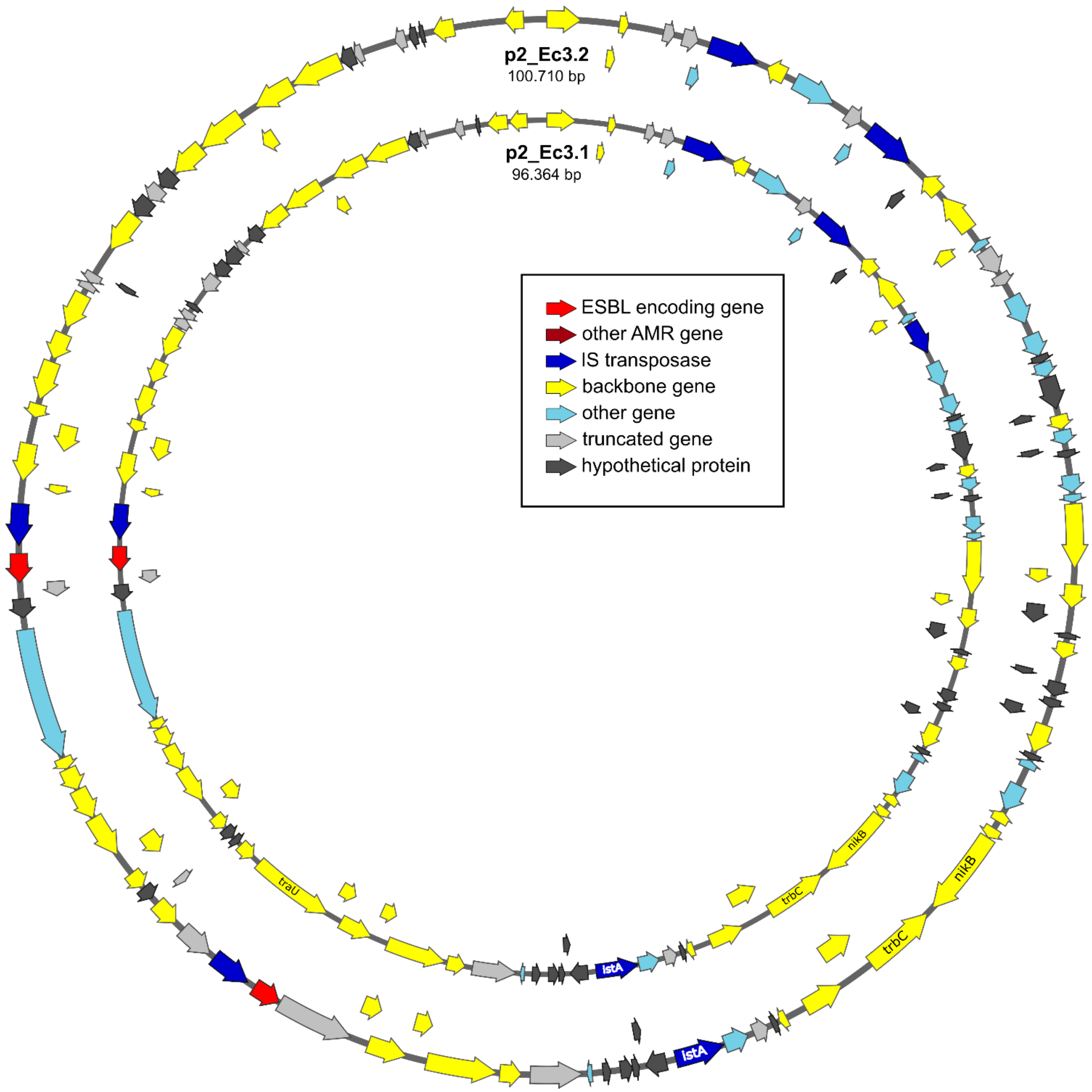

Supplementary Figure S3: Plasmid map pair III, annotated plasmids p2_Ec3.1 und p2_Ec3.2

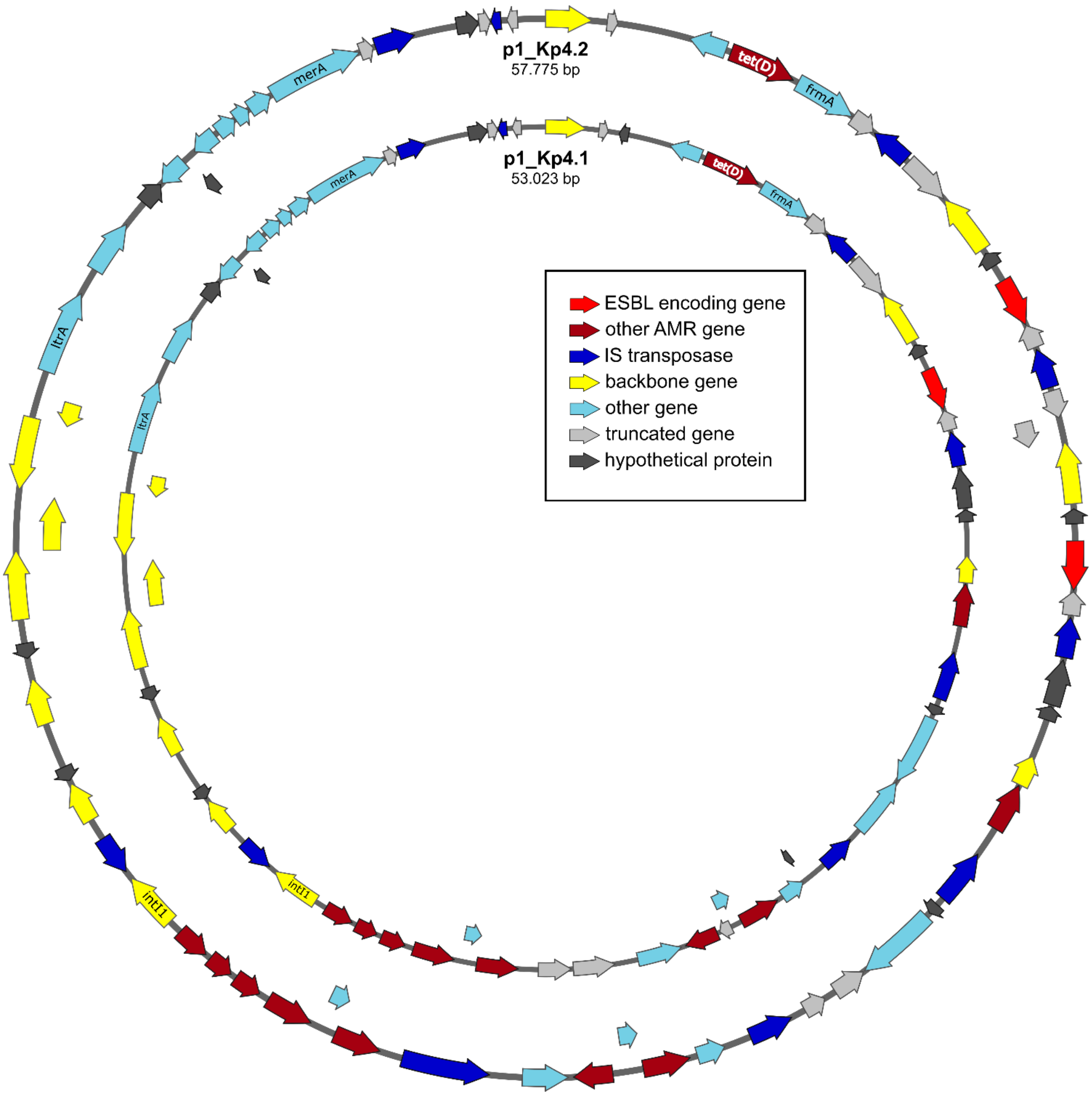

Supplementary Figure S4: Plasmid map pair IV, annotated plasmids p1_Kp4.1 und p1_Kp4.2

2 Supplementary Tables

Supplementary Table S1: Summary of the plasmid and transposon analysis, numbers in brackets after the genes indicates multiple gene copies.

plasmid	AMR genes	Mobile genetic elements (No. of copies)	Length of analysed transposons [bp]	truncated genes or gene products in the transponsons	transposon type
p3_Ec1.1	*bla*$_{\text{CTX-M-15}}$. *qnrS1*	IS*26* (2x)	10642	IS3, PinE site-specific DNA recombinase, transposase	composite transposon
p1_Ec1.2	*bla*$_{\text{CTX-M-15}}$ (2x), *qnrS1* (2x)	IS*26* (3x)	10650/10644	IS3, PinE site-specific DNA recombinase, transposase	
p2_Ec2.1	*bla*$_{\text{SHV-12}}$, *bla*$_{\text{SHV}}$-family, *qnrS1*	IS*26* (3x), IS*Kpn19*	5285/5795	IS26 (only in repeat 2)	composite transposon
p2_Kp2.2	*bla*$_{\text{SHV-12}}$ (2x), *bla*$_{\text{SHV}}$-family, *qnrB19*, *qnrS1*,	IS*26* (6x), IS*Kpn19*	5285/5284/5285	-	
p2_Ec3.1	*bla*$_{\text{CTX-M-14}}$	IS*Ecp1*, IS*Cro1* (2x), IS*KOx3*	3040	IS*5*, *traS*	unit transposon
p2_Ec3.2	*bla*$_{\text{CTX-M-14}}$ (2x)	IS*Ecp1* (2x), IS*Vsa5*, IS*Cro1* (2x)	3040/3040	IS*5*, *traS*, *traU*	

p1_Kp4.1	*arr-3*, *aac(3)-IId*, *aac(6')-Ib-cr5*, *aadA16*, *tet(D)*, *bla*$_{SHV-2}$, *dfrA27*, *sul1* (2x), *qnrB6*	IS*26* (3x), IS*6100*, IS*Kpn26*	5561	*ygbI*, MFS transporter	composite transposon
p1_Kp4.2	*arr-3*, *aac(3)-IId*, *aac(6')-Ib-cr5*, *aadA16*, *tet(D)*, *bla*$_{SHV-2}$ (2x), *dfrA27*, *sul1* (2x), *qnrB6*	IS*26* (3x), IS*6100*; IS*CR1*, IS*Kpn26*	5561/5562	*ygbI*, MFS transporter	

Supplementary Table S2: Chromosomal or plasmid-borne antimicrobial resistance genes in the studied isolates, in addition to Table 2 for the whole isolates and not only for the analyzed plasmids, numbers in brackets after the AMR genes indicates multiple gene copies.

**isolate**		**size [bp]**	**AMR genes**	**efflux pump genes**
Ec1.1	chromosome	4,735,599	*bla*$_{EC}$, *glpT*_E448K, *gyrA*_D87N, *gyrA*_S83L, *parC*_S80I, *parE*_E460K	*acrF*, *mdtM*
	p1asmid p1_Ec1.1	139,806	*aph(3'')-Ib*, *aph(6)-Id*, *sul2*	-
	p1asmid p2_Ec1.1	106,497	*mef(C)*, *mph(G)*	-
	p1asmid p3_Ec1.1	72,476	*bla*$_{CTX-M-15}$, *qnrS1*,	-
	plasmid p4_Ec1.1	19,115	-	-
	plasmid p5_Ec1.1	16,854	-	-

Ec1.2	chromosome	5,137,266	*bla*$_{EC}$, *cyaA*_S352T, *glpT*_E448K	*acrF, emrD*
	plasmid p1_Ec1.2	82,307	*bla*$_{CTX-M-15}$ (2x), *qnrS1* (2x)	-
Ec2.1	chromosome	5,220,160	*aadA1*, *bla*$_{EC}$, *bla*$_{TEM-1}$, *sul1*, *glpT*_E448K, *marR*_S3N, *pmrB*_E123D	*emrD, emrE*
	p1asmid p1_Ec2.1	118,989	-	-
	p1asmid p2_Ec2.1	57,462	*bla*$_{SHV-12}$, *bla*$_{SHV}$, *qnrS1*	-
	p1asmid p3_Ec2.1	6,077	-	-
	p1asmid p4_Ec2.1	5,728	-	-
Kp2.2	chromosome	5,246,115	*bla*$_{SHV-11}$, *fosA*, *oqxA*, *oqxB*, *ramR*_A19V	*emrD*
	p1asmid p1_Kp2.2	186,389	*bla*$_{SHV-1}$, *tet(D)*	-
	p1asmid p2_ Kp2.2	66,525	*bla*$_{SHV-12}$ (2x), *qnrB19*, *qnrS1*	-
	p1asmid p3_ Kp2.2	10,277	-	-
	p1asmid p4_ Kp2.2	59,234	*bla*$_{SHV-12}$ (7x), *qnrB19*, *qnrS1* (2x)	-

Ec3.1	chromosome	4,951,036	*bla*$_{EC}$, *glpT*_E448K, *gyrA*_D87N, *gyrA*_S83L, *parC*_S80I, *pmrB*_Y358N	*acrF*, *emrE*, *mdtM*
	p1asmid p1_Ec3.1	147,575	*aph(3'')-Ib*, *aph(6)-Id*, *bla*$_{TEM-1}$, *dfrA5*, *sul2*	-
	p1asmid p2_Ec3.1	96,364	*bla*$_{CTX-M-14}$	-
	p1asmid p3_Ec3.1	1,467	-	-
	p1asmid p4_Ec3.1	21,539	-	-
Ec3.2	chromosome	4,980,585	*bla*$_{EC}$, *glpT*_E448K, *gyrA*_D87N, *gyrA*_S83L, *parC*_S80I, *pmrB*_Y358N	*acrF*, *emrE*, *mdtM*
	p1asmid p1_Ec3.2	149,287	*dfrA5*	-
	p1asmid p2_Ec3.2	100,710	*bla*$_{CTX-M-14}$ (2x)	-
	p1asmid p3_Ec3.2	15,081	-	-
Kp4.1	chromosome	5,275,347	*bla*$_{SHV-1}$, *fosA*, *oqxA*, *oqxB*	*emrD*
	p1asmid p1_Kp4.1	53,023	*aac(3)-IId*, *aac(6')-Ib-cr5*, *aadA16*, *arr-3*, *bla*$_{SHV-2}$, *dfrA27*, *qnrB6*, *sul1* (2x), *tet(D*	-

Kp4.2	chromosome	5,252,719	*bla*$_{\text{SHV-1}}$, *fosA*, *oqxA*, *oqxB*	*emrD*
	p1asmid p1_Kp4.2	57,775	*aac(3)-IId*, *ac(6')-Ib-cr5*, *aadA16*, *arr-3*, *bla*$_{\text{SHV-2}}$ (2x), *dfrA27*, *qnrB6*, *sul1* (2x), *tet(D)*	-
	p1asmid p2_Kp4.2	32,531	*aac(3)-IId*, *ac(6')-Ib-cr5*, *aadA16*, *arr-3*, *bla*$_{\text{SHV-2}}$ (2x), *dfrA27*, *qnrB6*, *sul1* (2x),	-

Supplementary Table S3: VITEK®2 data of all bacterial isolates. MICs are given in mg/L. Data was interpreted according to EUCAST v. 13.1 and color-coded: yellow - susceptible; grey – intermediate; blue – resistant.

isolate	pair I		pair II		pair III		pair IV	
	**Ec1.1**	**Ec1.2**	**Ec2.1**	**Kp2.2**	**Ec3.1**	**Ec3.2**	**Kp4.1**	**Kp4.2**
ampicillin	>=32	>=32	>=32	>=32	>=32	>=32	>=32	>=32
piperacillin and tazobactam	<=4	<=4	<=4	64	<=4	<=4	<=4	32
cefuroxime	na	na	>=64	>=64	>=64	>=64	4	>=64
cefuroxime axetil	>=64	>=64	>=64	>=64	>=64	>=64	4	>=64
cefotaxime	8	>=64	>=64	>=64	>=64	>=64	<=1	8
cefpodoxime	na	na	>=8	>=8	>=8	>=8	4	>=8
ceftazidime	4	>=64	>=64	>=64	<=1	4	<=1	32
ertapenem	<=0.5	<=0.5	<=0.5	<=0.5	<=0.5	<=0.5	<=0.5	<=0.5
meropenem	<=0.25	<=0.25	<=0.25	<=0.25	<=0.25	<=0.25	<=0.25	<=0.25
gentamicin	<=1	<=1	<=1	<=1	na	na	na	na
ciprofloxacin	>=4	1	1	2	>=4	>=4	1	2
trimethoprim and sulfametoxazole	<=20	<=20	<=20	<=20	>=320	<=20	>=320	>=320
